# Improving pain‐related communication in children with autism spectrum disorder and intellectual disability

**DOI:** 10.1002/pne2.12076

**Published:** 2022-02-28

**Authors:** Rachel Fitzpatrick, Brian E. McGuire, Helena K. Lydon

**Affiliations:** ^1^ School of Psychology and Centre for Pain Research and Applied Behaviour Research Clinic National University of Ireland Galway UK

**Keywords:** autism spectrum disorder, challenging behavior, intellectual disability, pain, verbal operants

## Abstract

The communication of pain in individuals with co‐morbid Autism Spectrum Disorder and intellectual disability (ASD‐ID) is largely unexplored. The communication deficits associated with ASD‐ID can result in nonverbal behavior such as self‐injurious behavior, aggression, irritability, and reduced activity as a means to communicate that pain is present. The objective of this study was to determine whether a behavioral‐based educational intervention could increase the pain‐related communication of children with ASD‐ID who experience pain frequently. Specifically, the study aimed to determine if children with ASD‐ID can label the location of their pain or quantify pain severity and request pain relief. The sample included three children with ASD‐ID who experienced pain frequently. The intervention utilized educational materials and behavioral reinforcements and the intervention was conducted using a series of case studies. Pain was assessed daily by caregivers using the Non‐Communicating Children's Pain Checklist—Postoperative (NCCPC‐PV) and the ability of the individual to identify and express pain was recorded using the Wong Baker FACES Pain (WBFPS) Scale. Challenging behavior was recorded based on frequency count. The results indicated that all participants displayed the ability to independently respond to a question about how they were feeling by vocalizing the location of pain or indicating their level of pain on the WBFPS and requesting pain relief. The results suggest a role for behavioral‐based educational interventions to promote communication of pain in people with ASD‐ID.

## 1 INTRODUCTION

The International Association for the Study of Pain (IASP) defines pain as “an unpleasant sensory and emotional experience associated with, or resembling that associated with, actual or potential tissue damage”,^1^ pg. 1976). Chronic pain is generally considered to be pain that is present for at least 3 months. IASP recognizes that pain may be present even in those unable to reliably verbally communicate the presence of pain.^1^ People with co‐morbid Autism Spectrum Disorder (ASD) and intellectual disability (ID) may fall into this group. Indeed, this population is at increased risk of experiencing pain due to the presence of challenging behavior, including self‐injurious behavior and increased risk of accidental injury.^2^ Individuals with ASD and ID (ASD‐ID) may also present with co‐morbid health problems that may be associated with pain,^3^ including neurological, musculoskeletal, and gastrointestinal problems.

A small number of studies to date have looked at the prevalence and impact of pain in individuals with ID. Findings from caregiver reports have found wide‐ranging estimates of daily pain, ranging from 15%‐50% of people with ID.^4^, ^5^, ^6^ Pain impacts on the daily functioning of individuals with ID by inhibiting their ability to participate fully in day service activities,^6^ and negatively impacts on sleep,^6^ emotional well‐being, and quality of life.^7^

A recent study on the prevalence of pain (based on parental report), in a representative sample of children in the United States found that children with ASD showed a higher incidence (15.6%) compared with children without ASD (8.2%). However, the prevalence of pain was highest among children with ASD and developmental co‐morbidities (19.9%). The authors opined that underlying sensory sensitivities, comorbidity of conditions associated with pain such as cerebral palsy and gastrointestinal conditions, as well as more frequent medical procedures, could account for the elevated prevalence of pain.^8^

Self‐report is considered the “gold standard” in the assessment of pain; however, self‐report measures are not always accessible or feasible for use with individuals with ID and ASD.^9^ Furthermore, individuals with ASD‐ID are often reported to express their pain in “atypical” ways such as through self‐injurious behavior (eg, head‐banging and biting), aggression and changes in behavior such as irritability, low mood, reduced activity, appetite change, changes in sleep, or crying.^6^, ^10^, ^11^ Consequently, pain is frequently unidentified and ineffectively managed among individuals with communication impairments.^7^, ^12^

To date, only two studies have examined psychological interventions focused on pain in individuals with a diagnosis of ID, both using cognitive behavioral therapy to teach self‐management strategies for chronic pain.^13^, ^14^ These studies included individuals who were high functioning, with good verbal communication skills, and did not involve people with ASD and/or moderate ID. There is an ongoing need for research on interventions for people with more severe cognitive impairments and other complex presentations.^9^, ^15^

The ways in which children in general learn about the concept of pain is relatively under‐researched. The limited literature in this area suggests that age‐appropriate language should be used and that tasks such as drawing and vignettes should be used to allow children to communicate their concept of pain, such as asking children about how the person may be feeling and what the person may need to feel better or different.^16^ It is also known that children learn about how to express pain and how to manage pain through their observations and interactions with caregivers.^17^ Children can, in fact, influence the attention they receive from others depending on the strength of their display of distress and that of their facial cues.^18^

From their work on the concept of pain in children, Pate et al identified four themes labeled as follows: (i) “my pain related knowledge,” (ii) “pain in the world around me,” (iii) “pain in me,” and (iv) “communicating my concept of pain.” They concluded that the first three themes (ie, pain‐related knowledge, pain in the world around me, and pain in me) are inputs that combine in varying proportions to produce the output which is how a child communicates their individual concept of pain.

There is therefore a reasonable conceptual basis to assume that labeling pain in oneself (pain in me) and labeling pain in others and identifying how the person is feeling (pain in the world around me) or identifying what someone would need to feel better (pain‐related knowledge) are prerequisites in order for a child to reliably communicate their own pain and their pain management needs.

The current study aimed to use a behavioral‐based educational intervention to teach children with ASD and ID to communicate about their pain, by communicating the severity of pain, reporting the location of the pain, and requesting pain relief, using a series of case studies, in which the initial baseline was staggered and ongoing probes were taken to monitor for the emergence of the skill. Data were also gathered on challenging behavior and pain (as measured by the Non‐Communicating Children's Pain Checklist) to (i) identify if pain was present for each participant, (ii) utilize this information in order to facilitate skills teaching when pain was present (participants learning the skill of communicating pain), and (iii) determine if there was any reduction in challenging behavior in response to the ability to communicate pain.

## 2 METHOD

### 2.1 Inclusion criteria

The study was open to children ranging in age from 5‐18 years who (i) had a confirmed diagnosis of ASD (made by an interdisciplinary service), (ii) presented with co‐morbid ID (as confirmed by formal psychometric and functional assessment), (iii) presented with a delay in language function, (iv) never vocalized or reported pain, and (v) experienced daily or frequent pain based on *The Non‐Communicating Children's Pain Checklist‐Postoperative Version* (NCCPC‐PV;^19^ where a proxy‐reported score >6 indicates the presence of pain.

### 2.2 Participants

Three male children with a diagnosis of ASD and ID were recruited from a school for children with special educational needs. Participant 1 was aged 11 years and 4 months with a diagnosis of ASD, moderate level of ID, co‐morbid neurological conditions and limb amputation which resulted in him having a prosthetic limb. The *Vineland Adaptive Behavior Scales* ^20^ assessment indicated that the verbal behavior of this participant was at 2 years and 7 months while expressive language was at 24 months. He expressed about 5‐10 words verbally and could echo several complete words. Staff reported that they believed he experienced pain frequently from the prosthetic limb and from constipation. He had a history of self‐injurious behavior (ie, hand biting) and challenging behavior (ie, crying and refusal) and these behaviors were reported to occur when he experienced pain. A score of 22 was reported on the initial assessment of pain using NCCPC‐PV.

Participant 2 was aged 12 years and 8 months and had a diagnosis of ASD and ID. Participant 2 was not able to effectively communicate when he was in pain. His score on *The Psychoeducational Profile—Third Edition (PEP‐3*;^21^ indicated that his receptive language ability was at 1 year and 11 months, while expressive language was at 1 year and 8 months. Staff reports documented frequent experiences of pain related to dental issues and constipation. The participant had a history of aggressive behavior (ie, throwing objects, hitting, and shoving tables) and other possible pain‐related behaviors (ie, decreased activity, withdrawal from others, and difficult to distract or pacify). A baseline score of 20 was reported on the *NCCPC‐PV*.

Participant 3 was aged 11 years and 2 months with a diagnosis of moderate ASD and a moderate level of ID. Assessment on *The Wechsler Preschool and Primary Scale of Intelligence—Third Edition, WPPSI‐III* ^22^ indicated that verbal IQ was in the extremely low range. The participant suffered from constipation at times and staff estimated that pain may be present once a week. Participant 3 was unable to verbally communicate when pain was present and this would result in noncooperation, irritability, guarding part of body that would hurt and he would seek comfort through physical closeness. A baseline score of 18 was reported on the *NCCPC‐PV*.

### 2.3 Ethical considerations

Ethical approval was obtained from the academic institution hosting the research, which was conducted in compliance with the Declaration of Helsinki. Consent and assent were obtained before taking any baseline data.

### 2.4 Experimental design

A series of case studies were undertaken, in which the initial baseline was staggered across three participants and ongoing probes were used to determine whether an increase in the communication of pain (ie, communicating the severity of pain or reporting the location of pain, and requesting pain relief) in participants with ASD‐ID could be achieved using principles of applied behavior analysis.

Each of the four components of the intervention was introduced individually and sequentially: (1) label body parts on iPad, (2) label body parts on self, (3) label pain and score severity using the Wong Baker FACES Pain Scale, and (4) request pain relief. Following this, in‐situ training was implemented. The intervention was introduced in a staggered fashion across participants following achievement of each component for each participant.

### 2.5 Dependent variable

*Communication of pain* was the dependent variable and was defined as the participant: (i) either pointing to the Wong Baker FACES Pain Scale (WBFPS) to indicate the level of pain they were experiencing or (ii) verbally expressing the location of pain and (iii) verbally requesting pain relief. Data was gathered on all three behaviors. An *Independent Response* occurred in response to being asked “How are you feeling?” and was defined as an independent point to the WBFPS or verbally expressing the location of pain and an independent verbal utterance to request pain relief. An *Independent Initiation* occurred in the absence of any question about how the participant was feeling and was defined as an independent point to the WBFPS Scale or verbally expressing the location of pain, and an independent verbal utterance to request pain relief.

### 2.6 Measures

#### 2.6.1 Challenging behavior

Frequency of challenging behavior was collected by the therapist and teacher using an individualized data collection sheet to record the occurrence of challenging behavior. A “+” was marked for each instance of challenging behavior in order to gather a tally of the occurrence of challenging behavior across the day. A “−” was marked if no instances of challenging behavior occurred for the entire day. All three participants displayed different topographies of challenging behavior. Participant 1 had a history of self‐injurious behavior (hand biting) and crying when pain was present. *Hand biting* was defined as any instance where the child's teeth made contact with their hand with enough force to leave a visual mark on the skin and/or make hand bleed. *Crying* was defined as any instance in which the child engaged in loud vocalizations accompanied with tears when pain was present. Participant 2 had a history of aggressive behaviors (hitting others and throwing objects) when pain was present. *Hitting* was defined as any instance where the child would use their dominant hand with force and hit another person on their upper body (including arms, back, chest, and face). *Throwing objects* was defined as any instance where the child would pick up an object (chair, book, etc) that was in close proximity to them and throw it with force. Participant 3 has a history of noncompliance and irritable behaviors (crying) when pain was present. *Noncompliance* was defined as any instance where the child physically and/or verbally refused to follow an instruction or complete a task. The definition of crying was identical to that detailed for Participant 1.

#### 2.6.2 Pain

(a) The Non‐Communicating Children's Pain Checklist—Postoperative Version (NCCPC‐PV;^19^ is a modified version of the Non‐Communicating Children's Pain Checklist—Revised.^23^ The NCCPC‐PV is a pain measurement tool specifically designed for children with cognitive impairment and it has been shown to be valid and reliable for measuring pain in intellectual disability.^19^, ^24^ This study used the postoperative version of the measure as it was not possible to observe sleeping and eating across the duration of the study. This study used the English version to assess the level of pain at baseline and throughout the intervention. The English version is composed of 27 items divided into 6 subscales (vocal, social, facial, activity, body and limbs, and physiological). The measure is completed by parents/caregivers. Scores are obtained and calculated by adding the 27 items to obtain the total score. A total score of 6‐10 indicates a child has mild pain, and a total score of 11 or greater indicates a child has moderate to severe pain.^19^ The NCCPC‐PV was not used to make any medical decisions for the three participants.

(b) The Wong Baker FACES Pain Scale (WBFPS,^25^ is a measurement tool used to rate the severity of pain in children. The assessment tool contains a series of six round cartoon faces rating from 0 (no hurt) to 10 (hurts worst) beginning with a face that contains a smile representing “no hurt” to the sixth picture with a frown and tears coming from the eyes representing the “worst hurt”.

### 2.7 Baseline and probes conditions

**FIGURE 1:**
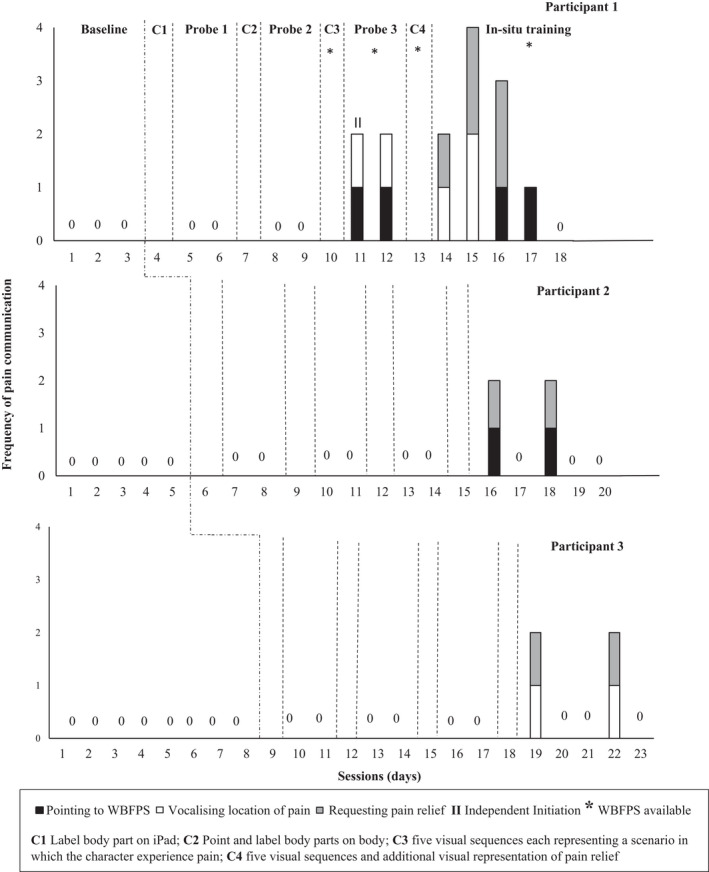
Frequency of communication of pain (pointing to WBFPS, vocalizing location of pain or requesting pain relief) for Participant 1, 2, and 3

During baseline, data were gathered on the communication of pain. All sessions consisted of a four‐hour period during the participant's typical school day. Probes were taken on two consecutive days following components (1) label body parts on iPad, (2) label body parts on self, and (3) label pain and score severity using the Wong Baker FACES Pain Scale. The introduction of subsequent components was based on stable responding on the communication of pain. All probe conditions were identical to baseline, with the addition of the WBFPS following Component 3. Following Component 3, the WBFPS was permanently placed on the participant's desk, this is denoted below in Figure 1 as an asterisk.

During baseline and across all probes, the teacher was working with the participant in their typical classroom environment, therefore, was aware if the participant engaged in any behavioral indicators of pain. The NCCPC‐PV was completed by the teacher at the end of each school day, and a score was calculated to quantify the level of pain behaviors. The NCCPC‐PV was used as a guide to observe pain‐related behaviors of all three participants within the baseline conditions. Frequency data was also collected on the occurrence of challenging behavior across a school day. At baseline and throughout the intervention phase, staff asked participants “How are you feeling?” when they believed the participant was experiencing pain based on the presence of challenging behavior or based on the NCCPC score.

### 2.8 Intervention phase

During the intervention phase, four target skills (labeling body parts on iPad, labeling body parts on self, identifying pain, and identifying pain relief) were identified as prerequisites to communicating pain and requesting (rather than just identifying) pain relief. Participants were assessed to verify if these skills were present and in cases where they were absent, the skill was taught to mastery. The mastery criteria for each component was 100% correct once or 90% correct on two consecutive trails. Training on the four components took place for 1 hour per session and was consistent for all three participants. Prior to commencing teaching sessions, each participant was presented with a choice board containing three preferred items and offered to select one item, which they would receive as a reinforcer during the teaching session. Each participant had individualized choices, token boards, and tokens.

#### 2.8.1 Component one

The first component of the intervention consisted of labeling body parts (head, arms, legs, back, and stomach) using a representation of a body on an iPad using the “Learn Body Parts” app. [Details on how to deliver training to teach this component can be obtained from the corresponding author on request].

#### 2.8.2 Component two

Component two was introduced, whereby the participant had to point and label the requested body part on themselves. The participant was presented with the instruction “show me ____” (eg, head), participants were required to respond by pointing to the body part on themselves.

#### 2.8.3 Component three

This component consisted of the presentation of five painful scenarios (ie, falling off a bike, playing football, hit head against a door, falling off a swing, and burning hand with hot water). Each scenario contained three pictures on flashcards outlining a story in which the character experienced pain, each scenario began with an image of a boy or girl doing an activity but without evidence of injury or pain. The participant was presented with one scenario at a time and then the WBFPS. During the teaching session, each participant was presented with all five scenarios (see Table 1).

**TABLE 1:** Description of the visual sequences and options for pain relief

Scenario	Event	Visual sequence	Pain relief option
1	Falling off a bike	A boy on his bike and smiling (no pain) The boy had fallen off his bike and was on the ground The boy was crying (tears) and that there was a visible cut on his knee from falling off his bike (ie, in pain)	*Band aid on knee* or cold pack for head
2	Injury while playing football	A boy holding a football in his right arm and smiling (no pain) The boy was in the park playing football with his friends The boy had been injured playing football. The picture shows that the ball had hit the boy's head and was he upset (ie, in pain)	*Cold pack for head* or cream for arm
3	Hitting head against a door	A boy walking into school and smiling (no pain) The boy had walked into a door and hit his head The boy had an injury to his head (bump on head which was large and red) and was visibly upset (ie, in pain)	Cream for arm or *cold pack for head*)
4	Falling off a swing	A boy on a swing and smiling (no pain) The boy had fallen off the swing The boy injured his arm (arm was bleeding) and he was visibly upset (ie, in pain)	*Band aid for arm* or cold pack for head
5	Burning hand with boiling water	A boy and a girl in a kitchen cooking (holding kitchen utensils, no pain) The girl had spilled hot water from a saucepan (steam coming from saucepan) onto her hand. Her hand was red to demonstrate a burn The girl had burnt her hand and was in pain (crying and holding her hand, that is, in pain)	Band aid on knee or *cream for hand*

Note

For pain relief options, the correct option is denoted in italics. Within the current study, the visual sequences were deemed to display a moderate level of pain. Therefore, a correct response was deemed to be a score of 6 or above on the Wong Baker FACES Pain Scale (WBFPS).

The therapist presented the scenario in conjunction with five questions (How is s/he feeling (at the start of the sequence)? What is s/he doing? What happened? Where does it hurt? How is s/he feeling (at the end of the sequence)?). Data was taken on three trials which related to answering “how is s/he feeling?” (at the start of the sequence), “where does it hurt?” and “how is s/he feeling?” (at the end of the sequence). When the researcher gave the instruction “how is the person feeling?” pointing at picture 1, the participant was then presented with the WBFPS to which they had to point to the appropriate face. The purpose of labeling “not hurt” (happy) at the start of the sequence was essential as it allowed the participant to differentiate between no pain and the presence of pain. Five trials were conducted on each scenario.

If the participant labeled the correct face using the WBFPS, verbal praise and a token were given to the participant to place on their token board. However, if the participant labeled an incorrect face (eg, suggesting that pain was present when none would be expected), an error correction procedure was used whereby the therapist touched and labeled the correct face and said “s/he is not hurt.” Praise and tokens were withheld for any incorrect responses. The scenarios were all presented in the same way for each participant. The visual sequences were designed to display moderate levels of pain to ensure that the participant selected 6 or more on the WBFPS.

#### 2.8.4 Component four

This component consisted of the presentation of the flashcards with an additional visual representation of pain relief (four‐picture sequence). This component was delivered in the same way as component three with each scenario being accompanied by five questions (How is s/he feeling (at the start of the sequence)? What is s/he doing? What happened? Where does it hurt? How is s/he feeling (at the send of the sequence)?). However, when the participant labeled the level of pain on the WBFPS, the therapist then asked the participant, “what do they need to feel better?”. The therapist presented two visual images as options after the instruction was given; one correct response and one incorrect response. The participant had to pick the correct picture and place it next to the four‐picture sequence. See Table 1 for pain relief options presented for each scenario.

If the participant selected the correct picture of pain relief, the therapist provided praise (eg, “yes, he needed a band aid”) and a token was given to the participant for him to place on the token board. However, if the participant picked the incorrect picture (ie, selected an ice pack for a cut on the arm instead of selecting a band aid, an error correction procedure was carried out by the therapist. The therapist stated “he needs a band aid” while pointing to the item. The therapist would then give the instruction “what does he need?” to which the participant had to pick the correct picture and place it next to the four‐picture sequence. Praise and tokens were withheld for any incorrect responses. Teaching continued until mastery was achieved.

### 2.9 In‐situ training

Individuals with ASD often have difficulty with skill generalization. To address this, in‐situ training was implemented for 5 days. The aim of in‐situ training was to transfer the use of the WBPFS and the visual supports for pain relief used during Component 4 to support participants to communicate their pain. Participants had continuous access to the WBPFS and a visual support containing images of their individualized pain relief regimen (eg, for participant 1, an image representing Vaseline cream and quiet space was attached to his desk). Data were also collected on level of pain using the *NCCPC‐PV* (for each 2‐hour period in the 4‐hour session) and any instances of challenging behavior were recorded.

Each participant's teacher was trained to deliver prompts, if required, when the participant may have been experiencing pain (ie, if a score of 6 or more on NCCPC‐PV was obtained or if challenging behavior (which was associated with pain) was present). The teacher posed the question, “How do you feel?”. If Participants did not emit an independent response to the question they were prompted using a gestural prompt (directing the participant to the Faces scale) and then presenting the participant with the visual images of the pain relief options available. If participants did not point to a visual representation of pain relief or verbally request pain relief, they were provided with a gestural and verbal prompt to request pain relief.

## 3 RESULTS

### 3.1 Intervention phase

#### 3.1.1 Component 1 and 2

All three participants correctly labeled and vocalized the five targeted body parts on the iPad (Component 1) and on themselves (Component 2). The participants had already acquired the skill of labeling their body parts, as they achieved 100% correct responding first time (see Table 2).

**TABLE 2:** Participants’ scores (number of correct responses) on labeling body parts and pain, identifying level of pain (Wong Baker FACES Pain Scale WBFPS), and requesting pain relief

	Participant 1	Participant 2	Participant 3
Component 1 (label body parts on iPad)	15/15	15/15	15/15
Component 2 (point and label body parts on body)	15/15	15/15	15/15
Component 3 (presentation of five visual scenarios depicting pain)			
Scenario 1	11/15, 15/15	13/15, 15/15	10/15, 15/15
Scenario 2	12/15, 15/15	13/15, 15/15	11/15, 15/15
Scenario 3	11/15, 15/15	15/15	14/15, 15/15
Scenario 4	15/15	15/15	15/15
Scenario 5	15/15	15/15	15/15
Component 4 (visual scenarios and additional visual representation of pain relief)			
Scenario 1	15/15	15/15	13/15, 15/15
Scenario 2	15/15	14/15, 15/15	14/15, 15/15
Scenario 3	12/15, 15/15	14/15, 15/15	14/15, 15/15
Scenario 4	12/15, 15/15	15/15	15/15
Scenario 5	15/15	15/15	15/15

#### 3.1.2 Component 3

All participants successfully labeled the location of pain in all five scenarios and quantified the severity of pain using the Faces Scale following 2 days of training.

#### 3.1.3 Component 4

All participants successfully labeled the location of pain on all five scenarios, quantified the severity of pain using the Faces Scale, and requested appropriate pain relief following 2 days of training.

### 3.2 Communication of pain

#### 3.2.1 Baseline

During the baseline phase, communication of pain was not observed for any of the three participants in a reliable or readily identifiable manner.

#### 3.2.2 Probes 1, 2 and 3

For Participant 1, no communication of pain was observed during Probes 1 or 2. During Probe 3, communication of pain was observed following Component 3. Participant 1 made one independent initiation by pointing to the WBFPS and vocalizing the location of pain and made one independent response by pointing to the WBFPS and vocalizing the location of pain. For Participant 2 and 3, no communication of pain was observed during probes 1, 2 or 3.

#### 3.2.3 In‐situ training

During in‐situ training, Participant 1 was observed to engage in requesting pain relief in addition to either vocalizing the location of pain or pointing to the WBFPS. On Day 1, he made an independent response by verbalizing the location of pain and requesting pain relief. On Day 2, he made two independent responses by verbalizing the location of pain and requesting pain relief and on Day 3, he pointed to the WBFPS on one occasion and requested pain relief on two occasions. On Day 4 he pointed to the WBFPS. Participants 2 and 3 engaged in independent responses on two of the 5 days. Participant 2 pointed to the WBFPS and verbally requested pain relief on both days. Participant 3 verbalized the location of pain and requested pain relief on both days (see Figure 1).

During in‐situ training, there were occasions when staff reported a score of above 6 on the NCCPC‐PV and prompted the participant to communicate pain; however, the participants did not always do so. However, results do show that participants engaged in communication of pain on days when their NCCPC‐PV scores were highest. For P1, for example, communication of pain was associated with NCCPC‐PV scores of 11, 14, 9 and 8. For P2 and P3, communication of pain was associated with NCCPC‐PV scores of 11 and 14, and 14 and 10, respectively.

### 3.3 Pain scores and challenging behavior

#### 3.3.1 Baseline

**FIGURE 2:**
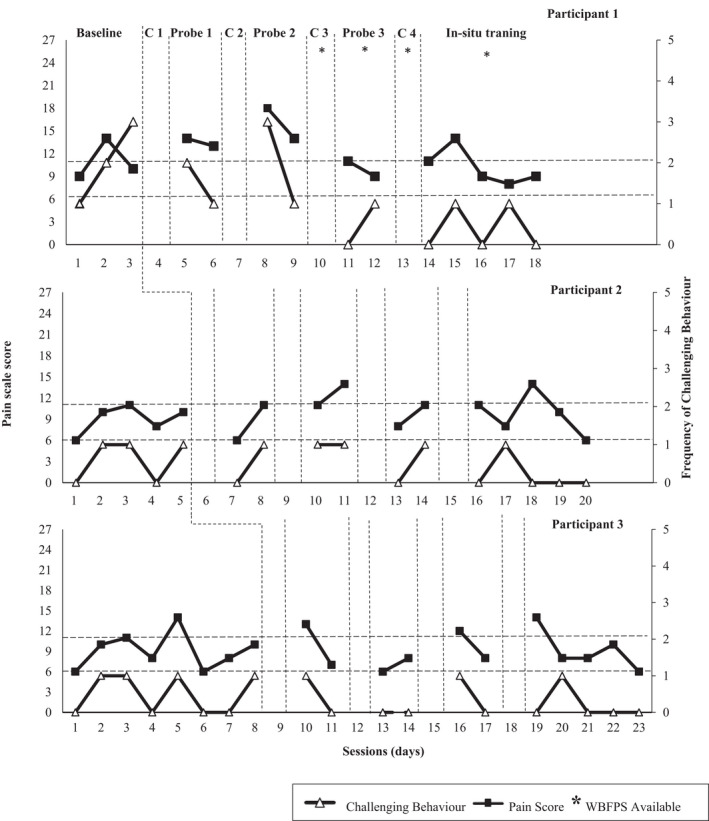
Pain scale score and challenging behavior for Participant 1 (top), Participant 2 (middle), and Participant 3 (bottom). The asterisks denote the presence of the WBFPS in the environment. The two dashed lines illustrate the NCCPC‐PV threshold (6) for identifying the presence of mild pain and (11) for identifying the presence of moderate to severe pain

During the baseline phase, pain was believed to be present for all three participants, as indicated by staff scores on the NCCPC‐PV. Participant 1 scored above the threshold (>6) on the NCCPC‐PV each day and displayed high levels of challenging behavior. Participant 1 experienced at least mild pain daily. Participant 2 and 3 scored above the threshold each day (indicating that they experienced at least mild pain daily). For Participants 2 and 3, it was noted that on days where participants scored 11 or higher on the NCCPC‐PV (ie, moderate to severe pain), challenging behavior was also evident. However, on days that they obtained a score of 10 or below (ie, mild pain), there was no occurrence of challenging behavior, indicating that there was a strong association between the presence of moderate to severe pain and the occurrence of challenging behavior (see Figure 2).

During the probes, Participant 1 scored above the threshold on the NCCPC‐PV each day and displayed high levels of challenging behavior, with the exception of Day 1 in Probe 3 (when he did not engage in challenging behavior). However, on this day he independently initiated communication of his pain. Similarly, Participants 2 and 3 displayed challenging behavior on days where participants scored 11 or above on the NCCPC‐PV.

#### 3.3.2 In‐situ training

During in‐situ training, Participant 1 continued to display the presence of pain with scores on the NCCPC‐PV above 6 daily. However, in contrast to baseline and probe conditions, challenging behavior was reduced, occurring only on two of the 5 days. In addition, the number of incidents of challenging behavior was lower than baseline (during which challenging behavior occurred on all 3 days).

Participant 2 displayed pain daily across the 5 days with a score of 6 or above on the NCCPC‐PV. Similar to Participant 1, challenging behavior was lower than baseline (during which challenging behavior occurred on 3 of the 5 days) with challenging behavior only present on one day.

Participant 3 was found to be experiencing pain daily across the 5 days with scores of 6 or above on the NCCPC‐PV. Similar to findings for Participant 1 and 2, challenging behavior was lower than baseline (during which it occurred on four of the 8 days), with its presence only recorded on one of the 5 days.

### 3.4 Treatment fidelity

The researcher used a fidelity checklist to ensure that the intervention was implemented consistently across all three participants. The fidelity checklist included a 19‐item checklist of each step of the intervention. The researcher delivered the intervention while a teacher/teacher's assistant observed the intervention and completed the fidelity checklist. Fidelity checks were taken for over 30% of sessions and was 100% for all training sessions.

### 3.5 Inter‐observer agreement

Inter‐observer agreement was assessed in all components of the study by a second observer. Both the first and second observer took data independently for all targeted responses. For participant 1, the inter‐observer agreement was 92.5% with a range of 80%‐100%, while for participant 2, inter‐observer agreement was 97.5% (range 90%‐100%), and for participant 3, inter‐observer agreement was 95% (range 80%‐100%).

## 4 DISCUSSION

This study aimed to increase communication of pain among three individuals with autism spectrum disorder and intellectual disability using a behaviorally‐based educational intervention by ensuring the presence of prerequisite skills (labeling and identifying) and using in‐situ training. All three participants learned how to report when pain was present, by reporting the severity of pain using the Wong Baker FACES Pain Scale or verbalizing the location of pain, and then requesting pain relief. The results showed that for all three participants, labeling body parts (Component 1 and 2) did not generalize to communication of pain for the participants themselves. Following teaching focused on identifying pain in others (Component 3), communication of pain increased for one of the three participants. In‐situ training involving prompts (gestural or verbal) was required to increase the communication of pain, in particular the request for pain relief, for all three participants.

Pain was present for all three participants across the experiment, as the NCCPC‐PV scores were above 6, indicating that they displayed behavioral indicators of pain on a daily basis throughout the study. During baseline and probes, it was noted that Participant 2 and 3 consistently displayed challenging behavior when a score of 10 or above was obtained on the NCCPC‐PV. Participant 1 displayed similar results with the exception of Day 1 in Probe 3. During this session Participant 1 independently initiated communication of his pain. These findings are consistent with Symons et al^26^ who reported that individuals with ASD‐ID are more likely to engage in challenging behavior when reported pain scores are higher. For individuals with ASD, challenging behavior is a known means to express their needs and in the current context, the NCCPC‐PV appears to be an appropriate method to capture the presence of pain by proxy as it correlated highly with the occurrence of challenging behavior.

During in‐situ training, the score from the NCCPC‐PV and the occurrence of challenging behavior served as antecedents to identify for staff, suitable opportunities to teach the communication of pain, given the higher probability of the presence of pain for the participants. During this phase of training, the findings indicate that participants engaged in communication of pain on days when their NCCPC‐PV scores were highest. It is possible that the experience of more severe pain may have motivated the participants to communicate their pain. Furthermore, pain‐related communication was associated with lower levels of challenging behavior which may indicate that an alternative response (the communication of pain and requesting pain relief) may have acted to suppress the occurrence of challenging behavior.

For all participants, the Wong Baker FACES Pain Scale was present in their environment consistently following Component 3. For Participants 2 and 3, the presence of the visual support (WBFPS) alone was not sufficient to increase the independent communication of pain. From in‐situ training, it is evident that teaching in context (ie, in the presence of pain) and prompting was required to support individuals with ASD‐ID to communicate the presence of pain. However, for Participant 3, he did not use the WBFPS to denote that pain was present, but rather vocalized the location of his pain. The behavioral indicators of the NCCPC‐PV can be helpful to educators, to identify when an individual may be experiencing pain, and thus, enabling them to capture opportunities to teach the person to communicate their pain.

The current findings highlight the issue of skills generalization for individuals with ASD. Despite all participants displaying the skills of labeling body parts, the presence of pain and the severity of pain in others (based on the visual sequences), this did not consistently generalize to personally‐relevant pain‐related communication. It is evident that in‐situ training was required to teach the communication of pain, in the presence of the occurrence of pain, to enable individuals with ASD‐ID independently communicate their pain in response to being asked how they are feeling. During the research, Participant 1 engaged in one independent initiation; however, more work is needed to determine how this skill can be reliably established in this population.

### 4.1 Limitations and future research

Although all three participants increased their communication of pain, there are some limitations to the study. For example, each of the participants had different locations of pain, and not all of the relevant body parts were included in the study. The study used the five most common locations of pain from McGuire et al’s^7^ study on pain in individuals with ID. For Participant 2, dental pain was problematic, however, this study did not target dental pain, and as a result, the participant never communicated dental pain but did communicate when he had gastric pain. Future research could incorporate individualized location of pain for each participant.

Similarly, the scenarios used within the current study did not represent the individualized pain experienced by each of the three participants. The scenarios were standardized depictions of pain in common situations. Future research could compare the use of standardized depictions of pain versus painful situations individualized to each participant.

A limitation of the current research is that the WBFPS and visual representation of pain relief were not used throughout the study. The WBFPS was introduced during Component 3 and the visual representations of pain relief were introduced during Component 4 and remained present on the participant's desk in all subsequent phases. Future research should ensure the WBFPS and visual representation of pain relief are used throughout the study. Furthermore, the study design could be enhanced by the inclusion of an additional probe following Component 4, prior to the in‐situ training.

As pain is a subjective experience, it is difficult to confirm its occurrence for individuals who have limited ability to communicate. During in‐situ training, there were occasions when staff reported a score of above 6 on the NCCPC‐PV and prompted the participant to communicate pain; however, the participants did not always do so. Tools which rely on behavioral indicators of pain are the most accessible options in applied settings. However, future research could explore the use of other physiological indicators to identify the presence of pain.

Further prevalence studies are also needed to determine the extent and nature of pain in people with ASD‐ID and to elucidate the most common behavioral indicators of pain so that functionally equivalent behaviors can be taught.

## 5 CONCLUSION

This study showed that in‐situ training was required in addition to the presence of the prerequisite skills of being able to identify and label pain, in order to develop pain‐related communication. The current study highlighted that for individuals with ASD‐ID, a visual prompt (ie, the presence of WBFPS) alone does not increase communication to report pain or request pain relief. Following in‐situ training, all participants displayed the ability to independently respond to a question about how they were feeling by vocalizing the location of pain or indicating their level of pain on the WBFPS and requesting pain relief. In addition, the current study supports the clinical utility of the NCCPC‐PV as a measure to assist staff in identifying the presence of pain in individuals with ASD‐ID, and thus assisting in identifying appropriate opportunities to teach the communication of pain.
